# Cultural selection shapes network structure

**DOI:** 10.1126/sciadv.aaw0609

**Published:** 2019-08-14

**Authors:** Marco Smolla, Erol Akçay

**Affiliations:** Department of Biology, University of Pennsylvania, Philadelphia, PA 19104, USA.

## Abstract

Cultural evolution relies on the social transmission of cultural traits along a population’s social network. Research indicates that network structure affects information spread and thus the capacity for cumulative culture. However, how network structure itself is driven by population-culture co-evolution remains largely unclear. We use a simple model to investigate how populations negotiate the trade-off between acquiring new skills and getting better at existing skills and how this trade-off shapes social networks. We find unexpected eco-evolutionary feedbacks from culture onto social networks and vice versa. We show that selecting for skill generalists results in sparse networks with diverse skill sets, whereas selecting for skill specialists results in dense networks and a population that specializes on the same few skills on which everyone is an expert. Our model advances our understanding of the complex feedbacks in cultural evolution and demonstrates how individual-level behavior can lead to the emergence of population-level structure.

## Teaser

Social networks can affect the emergence and spread of culture, but how does culture shape these social networks in turn?

## INTRODUCTION

Our species’ success in spreading through all biomes of the planet and transforming environments is a direct result of our ability to accumulate cultural traits, i.e., socially learned skills, technologies, beliefs, and ideas that build on existing traits. Transmission and accumulation of culture is an inherently social process. The amount and nature of culture that populations accumulate are expected to depend on the structure of social groups in which it evolves. The most studied aspect of population structure is its size. Theoretical and empirical studies suggest that the size of a population may affect how much culture it can accumulate, but the exact nature of the relationship remains strongly debated. Some models suggest that cumulative culture will increase with population size (*1*, *2*), while others find that the relationship might be mediated through population density (*3*) or be contingent on environmental conditions (*4*). Previous laboratory experiments report that larger groups maintained higher cultural complexity than smaller groups (*5*), and when faced with a complex task, groups accumulated beneficial solutions more readily than single individuals (*6*, *7*). However, there is also evidence for the opposite effect (*8*), and historical studies do not always detect a consistent relationship between measures of population size and cultural complexity (*4*, *9*). Parts of these contradictory findings might be explained through covariation in other aspects of social structure such as connectivity and migration between different populations (*10*). However, there is little theoretical work on how aspects of social structure other than population size and migration rates might affect cumulative culture.

Cultural information is transmitted where individuals interact with each other. Hence, this information flows along the edges of social networks, which are, in general, nonrandom and mediated by diverse factors and processes such as spatial heterogeneity (*11*), homophily (e.g., behavior matching) (*12*), or social inheritance (*13*). All of these lead to nonrandom opportunities for information to spread. Therefore, we expect the processes that structure social networks to also affect how culture is transmitted and accumulated in populations and thus the adaptive properties of cumulative culture. Here, we focus on the interplay between the fine-scale social structure of a population and its cumulative culture.

The connectedness of a population can be characterized by the average degree centrality of individuals (i.e., number of individuals they are connected to) and the average path length between pairs of individuals (i.e., the smallest number of links one has to cross to go from one individual to the other). In well-connected populations, individuals share links with many other individuals (high degree centrality) and are connected to all individuals by a small number of links (short path length). Here, information can spread faster and farther (*3*, *14*, *15*), a prediction that is borne out in humans (*7*, *16*) and nonhuman species (*17*–*20*). These networks might be optimal where group activity requires coordination, e.g., in regards to social norms (*21*) and rituals (*22*). Faster information transmission, however, is not universally adaptive for groups. Fast convergence on a single solution can short-circuit a thorough exploration of the solution space and cause a group to settle for suboptimal solutions (*23*, *24*). On the other hand, networks with less efficient information diffusion can increase the chance to collectively discover a global maximum (*23*, *25*), innovate a wider variety of solutions to a given problem (*23*, *26*), or retain more information in a collective memory-retrieval situation (*27*). The desirable network structure (from the group’s perspective) thus depends on the collective problem to be solved or the required diversity of solutions—a trade-off between cultural convergence and cultural diversity, swift coordination versus broad exploration (*24*, *26*, *28*).

The above discussion implies that populations facing ecological pressures that require different types of cultural adaptations might be expected to evolve different network structures. In particular, if the processes that affect how and with whom individuals form connections are heritable (genetically or culturally), then network structure can evolve under natural or cultural selection. The evolving network structure, in turn, will determine the nature of cumulative culture in the population, which can further affect the future evolution of network structure. While the effect of network structure on the dynamics of cultural traits is well studied, the reverse effect of cultural dynamics on network structure remains unexplored and, to the best of our knowledge, there is no prior work on the on-going feedback between the two. We show here that the continued “eco-evolutionary” feedback between cumulative culture and network structure can have unexpected consequences.

Our approach is based on a simple, yet generally applicable and realistic model of dynamic social networks (*13*), where individuals make connections either by inheriting them from their parents (or other role models) or randomly. The probabilities of making these two types of connections determine the average degree and whether the network is well-connected and has short path lengths or is highly clustered with long path lengths. Previous work has shown that co-evolution of these linking traits with social behaviors can lead to unexpected dynamics such as the collapse of cooperation due to its effect on network evolution (*29*). Here, we model the co-evolution of network structure with culture in a population where individuals socially acquire traits to cope with their environment. As social learning only occurs between connected individuals, an individual’s neighborhood affects what it can learn and so how it will cope with the environment, intrinsically linking network and cultural dynamics. Given that network topology affects information flow, and thus what individuals can learn, we expect that different network topologies emerge in response to different requirements on cultural knowledge. To test this, we compare two different worlds: A generalist world that favors individuals with a broad selection of skills [e.g., societies where each individual contributes in similar ways to the subsistence of the group (*22*)], and a specialist world that favors individuals with high proficiency in at least one skill [e.g., societies with high division of labor that allows specialization, which requires a lot of time engaging with only one trait (*30*–*32*)].

## RESULTS

### Model overview

We model a death-birth process on a binary, undirected network where, at each time step, a random individual is selected to die and another individual is selected to reproduce to replace them. In the first model, we use a static network where the newborn acquires the position of the dead individual in the network. Subsequently, we model dynamic networks using the social inheritance model by Ilany and Akçay (*13*). In this model, the newborn individual is integrated into the existing social network by forming a connection to its parent with certainty, to each individual that is connected to its parent with probability *p*_n_, and to each individual that is not connected to its parent with probability *p*_r_. Last, we add selection on *p*_n_ and *p*_r_ based on individually and socially acquired traits.

In all iterations of the model, the newborn acquires traits either through individual learning (trial-and-error) or through social learning (copying) from those individuals it shares a connection with. Individual learning happens with a probability relative to how many traits exist in the world (Eq. 1). Social learning happens with a probability relative to the squared frequency of the trait occurrence in the neighborhood (Eq. 2). The rationale for the quadratic function is that a trait needs to be sufficiently common in the neighborhood to be observed and/or sufficiently often performed so that the individual receives enough exposure and opportunities to learn the trait. The quadratic function, thus, lowers chances to acquire traits that are (locally) rare (see the Supplementary Materials for a version with linear scaling that yields qualitatively similar results). Thus, our learning model is akin to complex contagion dynamics (*14*). Successful learning of a trait adds the trait to the individual’s repertoire. Successful repeated learning of the same trait increases the individual’s proficiency for the trait. We add selection that is based on either the total trait repertoire size (generalist environment) or the highest trait proficiency achieved in a single trait (specialist environment). We also considered an alternative learning model where individuals make single observations at each period and have a finite memory for their more recent observations, which gives qualitatively the same results (see Materials and Methods and section S2).

Details of the model, including simulation conditions and parameters, are given in Materials and Methods. Below, we first consider fixed simple graphs and complex dynamic graphs where linking probabilities *p*_n_ and *p*_r_ are fixed and the same for every individual. Then, we let linking probabilities *p*_n_ and *p*_r_ be heritable and vary between individuals so that they evolve in response to their fitness consequences.

### Network effects on cultural diversity

**Fig. 1:**
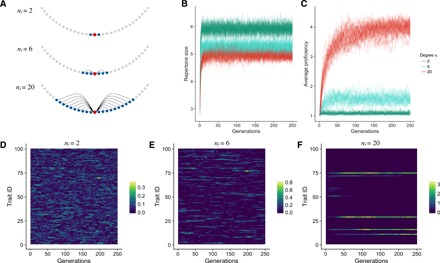
Increased connectivity leads to smaller trait diversity but higher trait proficiency. (**A**) Depending on the neighborhood size, *n_i_*, a focal individual (red), is connected to 2, 6, or 20 neighbors (blue). (**B** and **C**) Average repertoire size and average highest trait proficiency in populations with varying *n_i_*. Populations with larger *n_i_* (higher degree and shorter path length) have, on average, smaller repertoires and higher trait proficiency. (**D** to **F**) Example record of average proficiency of all traits in populations with varying *n_i_*. Highly connected populations (F) collectively have fewer traits but are more proficient at them than sparsely connected ones (D).

When we let culture evolve but keep the underlying social network fixed, we find that average degree affects repertoire size and trait proficiency at the individual level (Fig. 1, B and C) and trait diversity at the population level (Fig. 1, D to F). Individuals in denser networks have higher trait proficiency and smaller repertoires, whereas those in more sparsely connected groups have larger trait repertoires but lower proficiency. Repertoire size and trait proficiency are negatively related because of the trade-off between spending learning turns on acquiring or improving traits. In addition, because of finite population sampling effects, observing traits from more individuals is more likely to reinforce an existing skew in the trait distribution among neighbors. Consider a population where each individual has 1 of 100 possible traits. The probability that two random neighbors have the same trait is 0.01. For six neighbors, the probability that at least one trait appears multiple times grows to 0.14 and to 0.87 for 20 neighbors. As a result, we observe that dense networks have more skewed trait distributions and lower trait diversity than populations with sparse networks. In more connected groups, proficiency is higher because the lower trait diversity increases the chance to engage repeatedly with the same trait, which increases proficiency (see Eq. 2). In dense networks, each learning phase acts as a filtering process where rare traits will be learned less frequently, which further skews the distribution of traits on a population level leading to the loss of traits (Fig. 1F) and subsequently a steady accumulation of trait proficiency (Fig. 1B). In contrast, trait distribution is less skewed in sparsely connected networks. Individuals with low-degree centrality are more likely to have neighbors with a variety of traits, which lets the observer build up a larger repertoire. However, because individuals rarely engage repeatedly with the same trait, proficiency remains low. At the population level, this keeps average trait diversity high but prohibits proficiency increase.

While average repertoire size plateaus very quickly, average highest proficiency increases more slowly over the course of many generations (Fig. 1C). Because an observer’s trait proficiency cannot exceed the proficiency of the observed individuals through social learning alone, and given that innovations are rare, only populations with low trait diversity accumulate trait proficiency over time. Therefore, proficiency is higher in more connected populations.

### Dense networks have lower trait diversity and higher proficiency than sparse networks

**Fig. 2:**
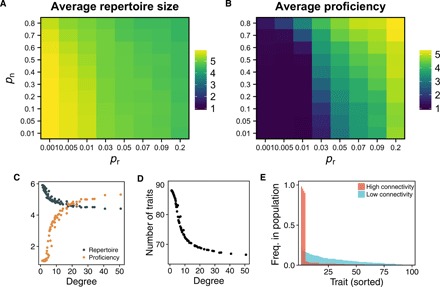
Effect of network topology onto cultural complexity. (**A** and **C**) Sparsely connected populations (low *p*_n_ and *p*_r_, low degree) have the largest individual repertoires, whereas well-connected populations (high *p*_n_ and *p*_r_, high degree) show the highest trait proficiency (**B** and C). (**D**) As average degree increases, the total number of traits known to the population decreases. (**E**) Moreover, the trait distribution for highly connected populations is skewed such that a few traits are known to almost the entire population, whereas in sparsely connected populations, traits are more evenly distributed such that almost all available traits are known to different subsets of the population [compare bottom left and top right corner in (A) and (B)]. All values represent population averages.

We observe the same patterns when we apply the cultural dynamics to complex networks with dynamic rewiring but with fixed probabilities of linking *p*_n_ and *p*_r_ (Fig. 2). For high *p*_n_ and *p*_r_ (high degree and clustering, short average path length; see section S3), individuals have high proficiency and small repertoire sizes, whereas for small *p*_n_ and *p*_r_ (low degree and clustering, long average path length), individuals have larger repertoires but lower proficiency (Fig. 2C).

Repertoire size and trait proficiency are more affected by the rate of random linking (*p*_r_) as compared to the rate of inheriting connections (*p*_n_) because average path length is most strongly affected by *p*_r_. Longer average path length creates isolation by distance, which prevents the spread of a single set of traits throughout the entire network and so avoids population-wide coordination on a few traits. As with fixed networks, we observe strong differences in trait diversity between tightly and sparsely connected networks, with a few very common traits in highly connected populations contrasting a widespread and distributed knowledge of traits in sparsely connected populations (Fig. 2, D and E).

### Specialists form efficient networks, whereas generalists form inefficient networks

**Fig. 3:**
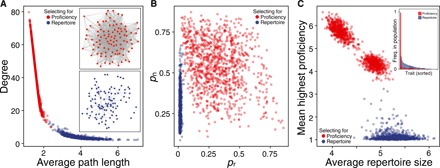
Specialist and generalist environments select for different network topologies. (**A**) Selecting for trait proficiency increases degree and decreases average path length, leading to dense networks (example in the top inset), whereas selecting for large repertoires decreases degree and increases path length, resulting in sparse networks (example in the bottom inset). (**B**) While generalist environments strongly select against random connections (*p*_r_), there is little selection against either linking parameter in specialist environments. (**C**) Selecting for generalists increases repertoire size while keeping proficiency at a minimum. Selecting for specialists reduces repertoire size only slightly but strongly increased individual proficiency. In addition, in simulations selecting for specialists, results are distributed in two clusters. Here, populations differ in the number of traits they converged on. Populations with higher proficiency converged on three traits, whereas populations with lower proficiency converged on four traits (note that average repertoire sizes are one trait larger due to trait innovation). Under specialist selection, trait distribution is highly skewed compared to generalist selection (inset).

Next, we let linking probabilities *p*_n_ and *p*_r_ evolve in response to selection for specialist (favoring high trait proficiency) or generalist (favoring large repertoire size) knowledge. When selection favors specialist knowledge, populations evolve to form dense networks with high average degree and short average path length (Fig. 3A). Conversely, selecting for generalist knowledge yields sparse networks with low-average degree and long average path length.

While, in environments favoring specialization, both linking probabilities appear to be under relatively weak selection, we find that there is strong selection against random connections when generalists are favored (Fig. 3B). This may at first appear counterintuitive, as random connections in a population with high trait diversity would allow individuals to be exposed to a completely different set of traits and, as a consequence, learn more different traits. However, we assume that successful social learning requires sufficient exposure to a trait (Eq. 2). Therefore, generalists have to form neighborhoods that trade off trait homogeneity to increase trait exposure with trait diversity to increase chances to observe different traits.

Selecting for generalists or specialists has a marked effect on the culture in each environment (Fig. 3C). Selecting for generalists results in populations with larger trait repertoire but low proficiency, and selecting for specialists results in populations with fewer traits but higher proficiency. Although there is a four- to fivefold gap in proficiency between specialists and generalists, the difference in average individual repertoire size is relatively small, less than two traits on average. This reflects a reduction in overall learning in the high-diversity environment of generalists where they do not get sufficient exposure to any single trait to attain high proficiency. In return, generalist populations as a whole carry many more traits at appreciable frequency (see inset in Fig. 3C), whereas specialists all learn the same few traits, resulting in a highly skewed trait distribution. This skewed trait distribution prohibits specialists from increasing their repertoire size, as all their neighbors converge to a small set of traits, whereas the broad trait distribution prohibits generalists from increasing their proficiency.

### Connectivity affects whether populations are generalists or specialists

The previous results show that generalists have a lower degree centrality than specialists. However, when degree centrality is externally dictated (high, intermediate, or low), individuals have to choose how many of their connections should be random links and how many should be socially inherited.

**Fig. 4:**
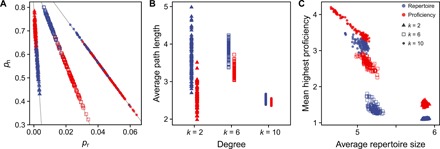
Trade-off between social inheritance and random connections depends on average connectivity. (**A**) Specialists benefit from convergence of traits in their neighborhood, which is achieved by increasing *p*_n_ (at low connectivity) or increasing *p*_r_ (at high connectivity). The opposite is the case for generalists who try to avoid trait convergence in their neighborhood. (**B**) This leads on average to shorter path lengths in specialists and longer paths in generalists (lines indicate possible combinations of *p*_n_ and *p*_r_ given degree *k*). (**C**) Populations are mainly made up of generalists at low connectivity and specialists at high connectivity. At intermediate connectivity, this is mediated by path length and clustering.

When degree centrality is small (*k* = 2), specialists are more likely to inherit connections than is the case for generalists (Fig. 4A). This is because specialists require a neighborhood with low trait diversity, and given that they always connect to their parents, they are more likely to find similar traits among their parent’s neighbors. For generalists, the opposite is the case. They are less likely to inherit connections and so increase the likelihood of learning from individuals with different trait sets. Therefore, average path length is longer in generalists than in specialists (Fig. 4B). In both cases, we find populations that are made of small clusters. However, generalists have a wider variety of traits present in those clusters than is the case for specialists (fig. S4, A and D), leading to slightly higher proficiency for specialists (Fig. 4C and fig. S5, A and D).

When degree centrality is intermediate (*k* = 6), we observe the reversed pattern. Here, generalists are more likely to inherit connections than is the case for specialists. At this level of connectedness, specialists benefit from trait convergence, which allows an increased trait proficiency (Fig. 4C). Generalists avoid trait convergence in their neighborhoods by increasing *p*_n_ and formation of loosely connected clusters, which increases average path length (Fig. 4B).

At an even higher degree centrality (*k* = 10), generalists cannot avoid some convergence of traits (fig. S4C), which leads to an overall increase in proficiency (Fig. 4C).

This shows that, depending on the average degree, a population will mostly be made up of either generalists (*k* = 2) or specialists (*k* = 10), while for intermediate degrees (*k* = 6), both states are possible and are mediated by average path length and clustering (Fig. 4C).

### When selection regimes change, specialists take longer to adapt than generalists

**Fig. 5:**
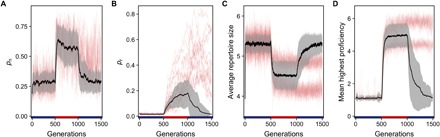
Populations more easily transition from generalists to specialists than the other way around. (**A** to **D**) Trajectory of linking probabilities (*p*_n_ and *p*_r_) and culture measures (repertoire size and proficiency) as selection regimes change from generalist (blue bars) to specialist (red bar) and back. Mean (black line) and SD (gray shading) for 100 repetitions are shown. Some simulations do not return to larger repertoires and lower proficiency after the specialist phase (red lines). Here, although *p*_n_ decreases after increasing in the specialist phase, (A) *p*_r_ keeps increasing even after selection is switched back to favoring generalists (B) (see main text for details). Note that the red lines remain on equilibria for repertoire size (C) and proficiency (D) that are identical to those shown in Fig. 3C and reflect the fact that for these parameters, populations may converge on three or four widely shared traits.

Switching between generalist and specialist environments reveals that the transition from sparsely connected populations with a broad distribution of traits occurs more readily than the transition from densely connected populations with highly specialized knowledge (Fig. 5). Subsequent to the switch to the specialist environment, average connectivity increases as *p*_n_ and *p*_r_ rise. This is followed by a decrease in average repertoire size and an increase in proficiency. Again, *p*_r_ starts to drift as populations specialize on a few traits (Fig. 5B), leading to increasingly dense networks. When the environment changes again and favors generalists, not all populations return to a less connected state, but instead *p*_r_ remains high (red lines in Fig. 5) and thus populations remain in a state of specialization. This is due to an echo chamber like effect where all individuals have an almost identical set of traits and so a newborn will learn those common traits and improve its proficiency, leaving little to no learning attempts to acquire more traits. Novel traits are still innovated, but they are rarely copied by others. A change can only happen in small, isolated clusters. Here, individuals with new innovations can escape the conformity pressure from the rest of the population. Eventually, given sufficient time, populations will return to a more sparsely connected structure with higher trait diversity.

## DISCUSSION

In this study, we combine cultural dynamics with evolving dynamic social networks to study how culturally mediated natural selection affects network structure. Our model highlights how selective effects of cumulative culture acquired from social connections determine both network structure and the diversity of cumulative culture at the individual and population level. We show that selection for generalist or specialist knowledge causes different network structures to emerge, with unexpected feedback onto population-level social and cultural dynamics. One might intuitively expect that selection for specialization might create internally clustered communities with sparse connections between them, whereas selection for generalists would favor more promiscuous connections to encounter a greater diversity of traits. We found the opposite effects: Selection for generalists produces sparsely connected networks with few random links, whereas specialists increase their random linking to generate densely connected networks.

### Cultural-evolutionary feedbacks between network structure and cumulative culture

Our counterintuitive results are driven by two factors, one at the individual level and the other at the network level. At the individual level, we assume that successful social learning requires repeated engagement with the same trait during the same learning episode. This assumption is encapsulated in Eq. 2, where the probability of learning a trait is quadratic in its frequency in the social neighborhood. It corresponds to the fact that learning in nature takes time and does not happen at the first contact with a novel trait (*33*–*36*). Our learning model is therefore akin to complex contagion transmission of behavior (*14*) and differs from previous cultural learning models that relied largely on simple infection contagion (*37*, *38*). A corollary of this learning model is that connecting with individuals who do not share many traits with each other depresses the overall rate of learning, since no single trait is likely to be repeatedly observed. Therefore, overall learning rates are highest when the traits of an individual’s connections overlap more. In ecological terms, this means that more learning happens when the beta diversity between connections, measuring the distinctiveness of individual repertoires, is low. The second factor is the cultural-evolutionary feedback (similar to eco-evolutionary feedbacks) from network structure to the population-level cultural trait diversity. This feedback means that densely connected populations converge to the same few traits, whereas sparsely connected ones display more network-level diversity through isolation by distance. This strong trade-off between population trait diversity and trait proficiency is mostly an emergent property. Although we also have (by necessity) a similar trade-off at the individual level, it is rather weak: Specialization reduces individual repertoire sizes only slightly.

When selection favors specialists, we initially observe the evolution of high social inheritance and networks with partially connected clusters that specialize on specific traits, but the high internal connectivity in these clusters leads to a loss of local trait diversity. Because of their success in specialization, these clusters grow, creating a skewed trait distribution at the population level where most individuals acquire the same few traits. As a result, even randomly formed links become likely to connect to an individual with similar traits to those connected through social inheritance. In turn, this reduces selection against random links, which increases average connectivity and further decreases trait diversity and skews trait distribution. The eventual outcome of this process is that almost all individuals will converge to an almost identical set of traits, and therefore the number and type of links become essentially neutral. Consistent with this finding, a recent study found increased shared knowledge among friends in hunter-gatherer societies (*39*). These nonkin friendship connections are formed early on in life and markedly increase network efficiency. Hence, they are equivalent to random connections (*p*_r_) in our model.

Conversely, selecting for generalists favors the ability to learn rare traits (perhaps individually innovated by a given connection). However, this is only possible when rare traits are relatively common within the local neighborhood of a focal individual, which favors low connectivity (as it decreases the total number of traits among the connections of a focal individual). This puts pressure on overall connectivity to decrease and, through isolation by distance, increases the overall cultural diversity of the population. One might think that the increased cultural diversity might “tempt” individuals to make more random connections to access new rare traits (which would undermine trait diversity at the population level), but this temptation is counteracted by the fact that being connected to individuals with too much diversity in traits depresses the overall learning rate.

We have modeled social tie formation in a heritable manner. Therefore, we model long-term changes to the topology of the social network. There is growing evidence showing that an individual’s network position can also change on much shorter time scales in response to their information status [reviewed in (*40*)]. A study on lemurs, e.g., found that knowledgeable individuals became more central in their group (*41*), whereas in *Drosophila*, more informed individuals were less central (*42*). There is a clear need for future models to address the effect of more rapid changes to social network topology on the diffusion and accumulation of cultural information.

### Exploration and coordination on social networks

Our results inform several important questions in the study of cultural evolution. For instance, much work has focused on how network structure affects the emergence and spread of social conventions and norms. While our model does not have direct social effects on payoff (i.e., the payoff of an individual depends only on its own trait), it nonetheless captures the same cultural-evolutionary dynamics underlying social norms and social information. Our results parallel recent experimental evidence showing that social conventions can emerge spontaneously and groups converge quickly on norms if the underlying social network is well connected (*43*). Another important question is how social network structure affects information aggregation. Social influence can bias individual estimates (*44*), but whether these biases improve or undermine the “wisdom of the crowd” depends on the underlying social network. A recent study found that decentralized groups became more accurate over time, whereas centralized groups, where central individuals have a disproportionally large effect on the collective estimation process, were more likely to increase in error (*45*). Furthermore, a recent laboratory experiment showed that a group’s performance in finding solutions to a complex problem can initially profit from dense information networks. However, the fast dissemination of successful solutions decreased exploration of the solution space and made well-connected groups more likely to settle for suboptimal local maxima (*23*). Our theoretical results are consistent with these findings and further imply that selection for proficiency can, in the long term, lock populations into well-connected networks, which, on one hand, might increase their ability to coordinate on a set of conventions but, on the other hand, diminish their capacity to explore or incorporate new information.

A related phenomenon is the spread of information and opinions on social media. Information that is shared in social media spreads in a complex contagion manner (*46*), where the likelihood that an individual will spread information increases monotonically with exposure (*47*). Previous research has connected homophily of connections (individuals tend to be connected with like-minded individuals) to the emergence of “echo chambers” where the same information gets shared over a network (*48*). Here, we show that the cultural-evolutionary feedbacks with complex contagion-like learning can produce convergence on the same few traits in well-connected networks even without explicit homophily.

An interesting area for studying cultural-evolutionary feedbacks is how scientific fields self-organize and explore different questions and methods. As research fields grow, networked communities of researchers emerge and the shape of these networks affects the spread of questions, study designs, and analytical methods (*49*). An interesting recent finding is that centralized research communities (where a few individuals are coauthors in many papers) are less likely to produce replicable results (*50*), while decentralized communities (where most papers have distinct coauthors) generate more robust and replicable results (*50*). These results are consistent with our model, which shows that short path length networks (i.e., centralized) tend to converge to a few traits and display strong priority effects where previously established traits are hard to overturn. In the scientific context, one would expect that the collective correction of scientific claims will be less efficient in centralized networks. Studying the feedback between evolving scientific networks and specialization into different scientific “traits” (*51*), as well as how evolving scientific communities interact with institutional and funding structures, will yield insights into the process of scientific discovery.

Our results also imply that structure emerges in the distribution of traits even in the absence of trait interactions, purely from the joint dynamics of social learning and social networks. For example, in groups selected for specialization, a few core traits will co-occur with each other in most individuals and will co-occur randomly with rare traits that are mostly individually learned. In human labor markets, e.g., the patterns of co-occurrence of traits or skills can have important effects on individual and aggregate performance and productivity (*52*). Although our model neglects many aspects of real labor markets, our results highlight how the distribution of socially learned job skills may be affected by co-evolving social networks, which, in turn, can affect the dynamics of the labor market. Likewise, the distribution of skills within and between individuals can affect how well teams solve problems (*53*) and therefore affect firm performance and economic productivity. An interesting direction for future research is how interactions between traits and the social network structure affect the network of skills and how this affects individual and group performance.

### Population size, networks, and cultural complexity

One of the ongoing debates in cultural evolution that we mentioned earlier is the role of population size (*1*, *2*, *10*) versus connectivity and mobility (*3*, *54*) in determining the cultural diversity and complexity. We find that larger populations maintain more traits (see section S8), but more connected groups achieve higher proficiency at the cost of overall trait diversity. We find that cultural-evolutionary feedbacks cause the transition between the generalist (highly diverse) and specialist (highly proficient) states to be highly nonlinear. At low connectivity, proficiency is low and trait diversity is high. When the network reaches a critical density (here, at an average degree of about 5), we find a sudden increase in trait proficiency. Because of the increased number of individuals learning and innovating along the same traits, trait proficiency increases. These dynamics can be interpreted as an increase in the “effective cultural population size” (for a few traits), which has been suggested as the main driver of the transition between Middle and Upper Paleolithic, marked not only by increased technological complexity but also by increased interconnectedness between groups (*55*). Consistent with our results, archeological analysis suggests that increased intergroup connectedness leads to decreased technological volatility (*56*).

Our simulations also show an upper limit for the number of traits an individual can acquire and, by extension, an upper limit of traits a population can carry. However, as part of cumulative cultural evolution, traits not only become more complex but also increase in number (*57*). To allow culture to continuously expand in our model, traits could directly affect learning, e.g., by making it easier to learn a trait [less costly; see (*58*)], or by directly affecting demography, e.g., by increasing carrying capacity (*55*, *59*).

## CONCLUSION

We find that network topology not only affects the diversity and accumulation of cultural knowledge but also is itself shaped by cultural selection due to a continuous eco-evolutionary feedback between social structure and culture. As we have shown, it is important to model individual-level interactions to understand this feedback. Recent technological advances allow us to gather detailed individual data (*39*). Further empirical research, especially long-term studies, will help to clarify the extent of cultural selection on human social networks. This not only will shed light on the origins of cumulative culture in our ancestors but also increase our understanding of human biology as a whole.

## MATERIALS AND METHODS

### The model

We modeled populations of *N* asexually reproducing individuals, with overlapping generations, in a world with *T* learnable cultural traits. These traits relate to, e.g., subsistence and social norms and, therefore, are relevant to an individual’s survival and reproduction. Traits were assumed to be equal in payoff and can be acquired independent from each other, as we were interested in how much and how well individuals learn but not what they learn.

Time was divided into rounds. Each round consisted of three steps: (i) one randomly selected individual leaves the population, (ii) a parent is selected and a new individual is added to the population, and (iii) the new individual acquires traits through innovation and copying. Restricting learning to a phase early in life was based on observations that children in hunter-gatherer societies acquire most skills before adolescence (*22*, *60*). During learning, the new individual has each 100 alternating asocial and social learning attempts, allowing her to either acquire new traits or improve proficiency in those she already has. At birth, an individual’s proficiency *l* is zero for all traits (i.e., *l_t_* = 0, *t* ∈ *T*). Trait proficiency increases through successful learning. When a new trait is acquired through asocial (innovation) or social (copying) learning, the proficiency of trait *t* increases from *l_t_* = 0 to *l_t_* = 1. As the individual’s repertoire size *R_i_* is the number of nonzero trait proficiencies *l*, trait acquisition increases *R_i_*. To become better at performing a trait, repeated engagement with it is required, as learning takes time (*33*–*36*). Therefore, proficiency increases ($l_{t}^{\prime }=l_{t}+1$) with each successful asocial or social learning attempt of the same trait. Because the number of learning turns is limited and attention to one trait limits attention to other traits, there is a trade-off between becoming good at a trait and learning many traits. Hence, trait proficiency and repertoire size are negatively related.

During a single learning episode, an individual first picks one trait either from all possible traits *T* at random (asocial learning) or from all traits performed in the individual’s neighborhood relative to their performance frequency in the neighborhood (social learning). The probability that an individual observes trait *t* in its neighborhood is given by $\pi_{t}=\frac{n_{i,t}}{R_{n_{i}}},$ where *n*_*i*,*t*_ is the number of *i*’s neighbors with trait *t* and *R_n_i__* is the sum of repertoire sizes of *i*’s neighbors. We based our assumption that an individual is not actively choosing a trait to learn on observations in traditional societies where children acquire knowledge through playful work (*61*) or by helping their parents with subsistence tasks (*36*, *60*, *62*). The traits with which they engage are those that are performed in their vicinity. After picking a trait, the individual attempts to acquire proficiency for this trait either through individual learning (which depends on a fixed innovation success probability γ) or social learning (which depends on a fixed copying success probability σ). We assume that social learning is more effective when an individual receives more exposure to a trait, i.e., if π_*i*,*t*_ is larger. More generally, we can express the probability of successful asocial learning of trait *t* during a single learning episode as

$$P_{I}(t)=\frac{1}{T}\gamma =\frac{\gamma }{T}$$ (1)

and for social learning as

$$P_{S}(t)=\pi_{t}({\sigma \pi }_{t})={\sigma \pi }_{t}^{2}$$ (2)

Equation 2 relates to Simpson’s index (*63*), a measure of diversity representing the probability that two randomly picked items of a population belong to the same type. Like Simpson’s index, *P_S_* behaves parabolically, where higher trait diversity is more strongly punished than lower diversity. Therefore, *P_S_*(*t*) is highest where all neighbors perform only trait *t* (section S1). However, as trait diversity in *i*’s neighborhood increases, trait exposure decreases, making it less likely to observe its performance sufficiently long to learn about it socially (fig. S3B).

With Eq. 2, we model complex contagion (*14*) as the probability of observing a trait twice, whereby it does not matter whether the trait is observed from the same individual or from different individuals. This discounts the single observation of a trait with its square. Eq. 2 shows that acquiring a trait socially is more likely if social learning is easy (large σ), if trait *t* is common among neighbors (large *n*_*i*,*t*_), and if neighbors have few traits (small *R_n_i__*). Furthermore, we assumed that an individual cannot surpass the proficiency of the observed individuals and thus *P_S_*(*t*) = 0 where all neighbors have proficiency equal to or less than that of the individual *i* for trait *t*.

In our model, choosing a trait and attempting to learn that trait happens during the same learning episode. An alternative model would be that the two events occur at different points in time. To test whether our main results hold up to alternative assumptions about social learning, we developed a second learning model (see section S2). In this version, individuals have a separate memory to store traits they previously engaged with but have not yet attempted to acquire. We allowed individuals to remember traits they encounter for up to *m* learning episodes, after which they would be replaced by the most recent trait the individual engaged with. We found that the trade-off between trait diversity and proficiency remained unchanged as long as memory capacity was limited. Larger memory capacity reduces overall trait proficiency, as it reduces the coordinating effect of complex contagion.

Subsequent to the learning phase, we calculated an individual’s lifetime success score or payoff *W*. Its magnitude depends on whether the individual acquired traits according to its environment. Individuals face one of two environments. In the generalist world, individuals benefit from acquiring a variety of traits and so an individual’s payoff is equivalent to its repertoire size, *W_i_* = *R_i_*. In contrast, in the specialist world, individuals benefit from becoming highly proficient in one trait. Here, an individual’s payoff is equivalent to the highest trait proficiency in its repertoire, *W_i_* = max (**L***_i_*), where **L***_i_* is a vector of *i*’s proficiencies. The two environments can represent a variety of contexts, such as foraging. The generalists might forage on ephemeral, easy to handle but highly diverse resources, whereas the specialists might forage on stable, less diverse but hard to handle resources.

A new simulation round starts with the removal of a random individual. A survivor was selected as a parent to replace the individual relative to its payoff *W_i_*.

### Model iterations

#### Topology effects on culture

To establish a baseline for the effect of network topology on cultural dynamics in our model, we began with a set of static, regular networks (ring). We used different neighborhood sizes (1, 3, and 10) to alter topology (degree, clustering, and average path length) and measure its effect on average repertoire size and average highest proficiency (Fig. 1).

In all subsequent simulations, we considered complex dynamic networks. This method mimics real-world networks and allows the dynamical formation of locally and globally clustered networks in response to different selective regimes (*13*). In complex networks, a new individual inherits two genes from its parent: *p*_n_ (probability to form connections with the neighbors of the parent) and *p*_r_ (probability to form connections with other individuals that are not connected to the parent). Mutation occurs with probability μ = 1, whereby mutated values are drawn from a normal distribution centered around the parent’s value with SDs of 0.1 and 0.01 for *p*_n_ and *p*_r_, respectively. We also ran simulations with lower mutation rates (μ = 0.01) and found that the results hold (section S6). While in the main text, we assumed that connections can be formed at no costs, we also ran simulations where each connection incurs a cost and found that costs can turn populations into generalists even if they are under specialist selection (section S7).

To determine the effect of topology on culture in dynamic, complex networks, we let networks dynamically rewire but keep *p*_n_ and *p*_r_ fixed throughout the simulation (Fig. 2). For both static, regular and dynamic complex graphs, selection was neutral and reproduction was random. Thus, the spread of cultural traits was only affected by network topology.

#### Co-evolution of network topology and culture

Next, we let both cultural knowledge and linking probabilities (*p*_n_, *p*_r_) evolve freely in response to the generalists or specialists environment (Fig. 3 and see section S5 for time series).

#### Cultural response to enforced network degree

In nature, degree centrality might be more strongly affected by external factors than cultural selection. For example, sparse networks might be the result of high costs to form and maintain social connections. Networks might also be dense because of social norms or simply the spatial distribution of individuals. In both cases, this can lead to suboptimal network densities. In this iteration, we investigated the trade-off between socially inherited and random connections when average degree is fixed. We ran simulations, where *p*_n_ and *p*_r_ were coupled, as to achieve a preset degree centrality *k* (see section S4 for calculations). A newborn still inherits *p*_n_ from its parent; however, *p*_r_ is based on a linear function with an inclination that depends on degree *k*. Should an individual form more than *k* connections, *k* − 1 connections were randomly chosen (one connection remains for the parent) and the rest were discarded. We simulated populations with low, intermediate, and high connectivity (*k* ∈ 2,6,10) for both environments (Fig. 4).

#### Cultural response to shifting environments

In a final set of simulations, we switched between both selective environments and observed the changes in topology and cultural repertoire (Fig. 5).

### Parameters

If not stated otherwise, we ran all simulations with *N* = 100 and *T* = 100 (see section S8 for additional values) for 1000 generations (*N* death-birth events, with data being averaged over the last 200 generations) and 100 repetitions, with mutation rate μ = 1, innovation success rate γ = 0.01, and social learning success rate σ = 0.75 (see section S9 for additional values). Complex networks were initialized with *p*_n_ = 0.1 and *p*_r_ = 0.01. To compare differences in cultural knowledge between populations, we recorded the average repertoire size and mean highest per individual trait proficiency. To compare networks, we recorded degree centrality, local clustering, and average path length. Degree centrality, a measure for connectedness, is the average number of connections an individual shares with other individuals (higher degree centrality signifies more connections between individuals). Local clustering (or transitivity) is the probability that an individual’s neighbors share a connection with each other. It is a measure for how close a neighborhood is to being a clique (fully connected). Average path length is the mean number of steps along the shortest paths between any pair of individuals in a network. It is often used as a measure for information transmission efficiency in a network.

## Supplementary Material

http://advances.sciencemag.org/cgi/content/full/5/8/eaaw0609/DC1

Download PDF

## SUPPLEMENTARY MATERIALS

Supplementary material for this article is available at http://advances.sciencemag.org/cgi/content/full/5/8/eaaw0609/DC1 Section S1. Social learning success probability Section S2. An alternative social learning model Section S3. Network metrics for fixed values of *p*_n_ and *p*_r_ Section S4. Coupling *p*_r_ to *p*_n_ to limit degree centrality Section S5. Time series for simulations with evolving *p*_n_ and *p*_r_ Section S6. Low mutation rate Section S7. Connection costs Section S8. Varying population size and trait number Section S9. Varying innovation and social learning success rate Fig. S1. The effect of increasing memory on trait repertoire and highest skill level. Fig. S2. If memory size is limited, then the two different social learning algorithms are qualitatively the same. Fig. S3. The effect of complex contagion on social learning dynamics, and of linking parameters on network characteristics. Fig. S4. Distribution of common traits depends on average connectivity. Fig. S5. Trait proficiency depends on the level of trait convergence and connectivity. Fig. S6. Trajectories for linking probabilities *p*_n_ and *p*_r_ averaged over all simulation runs for all three selection regimes (neutral, generalist, and specialist). Fig. S7. Results displayed as in Fig. 2 of the main text but with mutation rate μ = 0.01. Fig. S8. Adding a cost per connection reduces average degree in specialists, whereas generalists are less affected. Fig. S9. Added connection costs. Fig. S10. Varying the number of traits and individuals in a population. Fig. S11. Increasing population size also increases trait diversity in the population. Fig. S12. Varying innovation and social learning success rate. Reference (*64*)
