# Supplementary material for: Cultural selection shapes network structure
Source: Sci Adv. 2019 Aug 14;5(8):eaaw0609. doi: 10.1126/sciadv.aaw0609 (PMC6693906; doi:10.1126/sciadv.aaw0609)
Supplement: http://advances.sciencemag.org/cgi/content/full/5/8/eaaw0609/DC1 [file supp_5_8_eaaw0609__index.html]

Science Advances | Science AdvancesAAASSearchScience AdvancesMenu

## Supplementary Materials

**This PDF file includes:**

- Section S1. Social learning success probability
- Section S2. An alternative social learning model
- Section S3. Network metrics for fixed values of *p*n and *p*r
- Section S4. Coupling *p*r to *p*n to limit degree centrality
- Section S5. Time series for simulations with evolving *p*n and *p*r
- Section S6. Low mutation rate
- Section S7. Connection costs
- Section S8. Varying population size and trait number
- Section S9. Varying innovation and social learning success rate
- Fig. S1. The effect of increasing memory on trait repertoire and highest skill level.
- Fig. S2. If memory size is limited, then the two different social learning algorithms are qualitatively the same.
- Fig. S3. The effect of complex contagion on social learning dynamics, and of linking parameters on network characteristics.
- Fig. S4. Distribution of common traits depends on average connectivity.
- Fig. S5. Trait proficiency depends on the level of trait convergence and connectivity.
- Fig. S6. Trajectories for linking probabilities *p*n and *p*r averaged over all simulation runs for all three selection regimes (neutral, generalist, and specialist).
- Fig. S7. Results displayed as in <Fig. 2 of the main text but with mutation rate μ = 0.01.
- Fig. S8. Adding a cost per connection reduces average degree in specialists, whereas generalists are less affected.
- Fig. S9. Added connection costs.
- Fig. S10. Varying the number of traits and individuals in a population.
- Fig. S11. Increasing population size also increases trait diversity in the population.
- Fig. S12. Varying innovation and social learning success rate.
- Reference (*64*)

Download PDF

**Files in this Data Supplement:**

- Adobe PDF - aaw0609\_SM.pdf
