# Supplementary material for: Cultural selection shapes network structure
Source: Sci Adv. 2019 Aug 14;5(8):eaaw0609. doi: 10.1126/sciadv.aaw0609 (PMC6693906; doi:10.1126/sciadv.aaw0609)
Supplement: Download PDF [file aaw0609_SM.pdf]

Science Advances
AAAS

advances.sciencemag.org/cgi/content/full/5/8/eaaw0609/DC1

Supplementary Materials for

Cultural selection shapes network structure

Marco Smolla* and Erol Akçay

*Corresponding author. Email: smolla@sas.upenn.edu

Published 14 August 2019, *Sci. Adv.* **5**, eaaw0609 (2019)
DOI: 10.1126/sciadv.aaw0609

This PDF file includes:

Section S1. Social learning success probability
Section S2. An alternative social learning model
Section S3. Network metrics for fixed values of p_n and p_r
Section S4. Coupling p_r to p_n to limit degree centrality
Section S5. Time series for simulations with evolving p_n and p_r
Section S6. Low mutation rate
Section S7. Connection costs
Section S8. Varying population size and trait number
Section S9. Varying innovation and social learning success rate
Fig. S1. The effect of increasing memory on trait repertoire and highest skill level.
Fig. S2. If memory size is limited, then the two different social learning algorithms are qualitatively the same.
Fig. S3. The effect of complex contagion on social learning dynamics, and of linking parameters on network characteristics.
Fig. S4. Distribution of common traits depends on average connectivity.
Fig. S5. Trait proficiency depends on the level of trait convergence and connectivity.
Fig. S6. Trajectories for linking probabilities p_n and p_r averaged over all simulation runs for all three selection regimes (neutral, generalist, and specialist).
Fig. S7. Results displayed as in Fig. 2 of the main text but with mutation rate $\mu = 0.01$.
Fig. S8. Adding a cost per connection reduces average degree in specialists, whereas generalists are less affected.
Fig. S9. Added connection costs.
Fig. S10. Varying the number of traits and individuals in a population.
Fig. S11. Increasing population size also increases trait diversity in the population.
Fig. S12. Varying innovation and social learning success rate.
Reference (*64*)

Supplementary Material

Section S1. Social learning success probability

In our model individuals successfully acquire a trait socially with probability

$$P_S(t) = \sigma \pi_t^2 \tag{3}$$

where $\pi_t = \frac{n_{i,t}}{R_{n_i}}$, i.e. the number of neighbours with trait t divided by the total number of traits exhibited in i's neighbourhood. We use a quadratic function in eq. 3, because we assume that an individual needs to observe a trait in its neighbourhood at least twice to learn it successfully. Figure S3A shows how the probability for successful social learning $P_S(t)$ changes as π_t moves away from 1. In fig. S3B we calculated $P_S(t)$ for different number of neighbours. It shows that acquiring a trait socially is most likely when all neighbours exhibit trait t, but quickly decreases as the trait becomes more rare in i's neighbourhood.

Section S2. An alternative social learning model

In the main text, we describe a model where learning is abstracted into learning episodes. An individual picks a trait relative to the frequency in its (social) environment and the probability that (social) learning is effective. Subsequently, the individual attempts to acquire proficiency for this trait through (social) learning. Here, we describe an alternative version of our model where these two steps may happen at different points in time.

Model Similar to the model described in the main text, during social learning an individual i observes a trait t with probability $P_s = \sqrt{\sigma}\pi_{i,t}$. If successful, the trait is added to the individual's 'memory.' If the trait already exists in the memory, we assume that the individual will learn that trait (i.e., learning a trait still requires two hits, but the first hit can be 'saved' in the memory between learning episodes). We assume that if an individuals' memory is full, the most recently observed trait drives out the oldest observed trait. This introduces a new parameter to the model, memory size. We considered memory to either be infinite (all encounters will be remembered), or finite (only the last m learning episodes are memorised). We ran simulations for $m \in \{1, 2, 10, T\}$, where $T = 100$ is the total number of traits in the world, corresponding to unlimited memory.

Note, in the model reported in the main text an individual i's proficiency for trait t increases with probability $p_s = \sigma\pi_{i,t}^2$, where σ is the overall success probability of social learning. Here, successful learning would be proportional to σ^2 (as increasing trait proficiency happens with probability P_s^2). We avoid this by using $\sqrt{\sigma}$ instead. Furthermore, since we now allow only a single observation per learning bout, the scale of the learning period doubles compared to our base model.

Results Figure S1 shows results for these experiments in comparison to the results we report in the main text (labelled 'quadratic'). If there is no restriction to the memory (labelled 'memory') individuals have generally much larger repertoire sizes (fig. S1c) than those reported in the main text. As predicted, we find that specialists have lower trait proficiency (fig. S1c). This is because when memory is unrestricted, individuals have a higher chance to acquire traits from their neighbours even if those traits are relatively rare. Therefore, specialists spend more learning turns improving the proficiency of a larger set of traits, which, in turn, keeps trait proficiency low.

An interesting result from simulations without memory restrictions is that generalists have much shorter path lengths. In the main text, we state that generalists avoid random connections (p_r) to circumvent trait convergence in their neighbourhood, which would lead to a smaller trait diversity and smaller repertoire sizes. Here, however, populations retain high trait diversity and so individuals increase their repertoire sizes by forming more connections. Being close to others now increases trait diversity without leading to trait convergence. This leads to relatively short path length and high degree and with a wide range of random connections in generalists (S1a,b).

Finally, as we decrease m our results become more similar to the model reported in the main text. Smaller m lead to higher trait proficiency in specialists, but also to smaller trait repertoires (S1c). The smaller repertoires also reduce the overall trait diversity in the population (S1d). Finally, where $m = 1$ we yield qualitatively similar results to those reported in the main text (fig. S2). If memory has only one slot an individual only acquires a trait if it successfully engages with it in two consecutive learning episodes, which is the same as raising the probability to the power of two.

Conclusions These results show that our results and the mechanism behind them are robust to reasonable alternative assumptions about social learning. In particular, the tradeoff between trait diversity and proficiency is there regardless of the learning model, or memory size. Larger memory sizes allow individuals to learn more traits counter-intuitively reduce proficiency, and in the limit, remove any difference between specialist and generalist populations. As long as memory is limited, however, the tradeoffs and mechanisms we identified in our original model hold qualitatively.

Section S3. Network metrics for fixed values of p_n and p_r

We systematically vary the values of p_n and p_r and observe the resulting network measures, i.e. degree, average path length, and local clustering. Degree centrality and local clustering are highest for large p_n and p_r, where all individuals are well-connected (Fig. S3C,D). However, clustering is also high where well-connected clusters are detached from each other (high p_n and low p_r). Average path length is longest for small p_r. However, for very small p_r well-connected clusters become detached, which leads to a sudden drop in path length (Fig. S3E). For larger values of p_r path length decreases again due to long distance links.

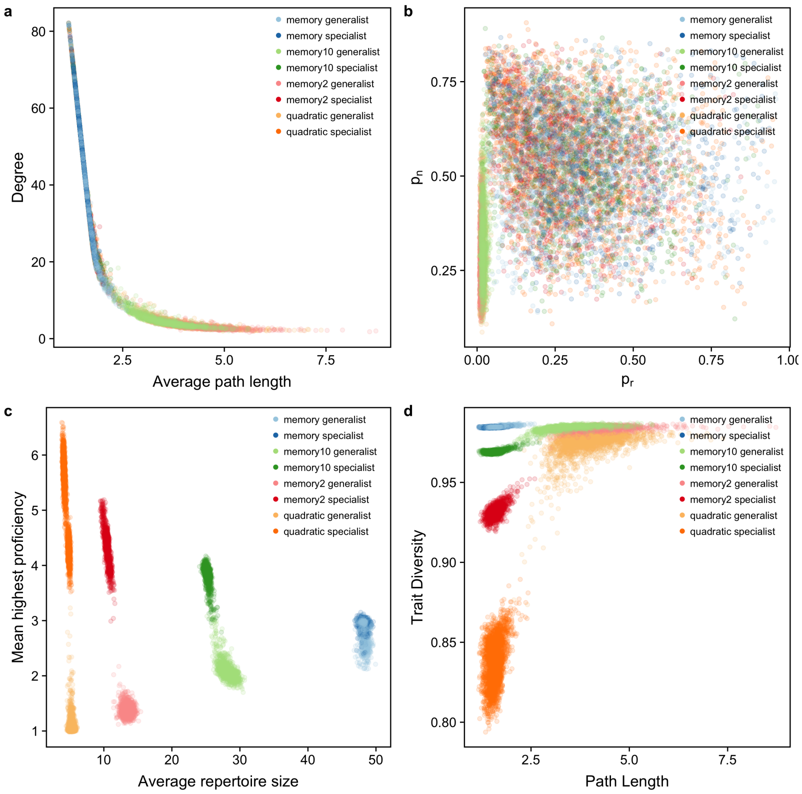

Fig. S1. The effect of increasing memory on trait repertoire and highest skill level. Results from simulations where repeated successful engagement with a trait throughout different learning episodes is stored in an individual's memory. Data shown are for memory size $m \in \{2, 10, T\}$ (labelled, 'memory2', 'memory10', and 'memory' respectively). For comparison, we also include the results from the main text (labelled 'quadratic'). Results for simulations with selection for proficiency ('specialists') and repertoire size ('generalists') are shown in darker and lighter shading. Parameters were the same as reported in the main text for Fig. 3.

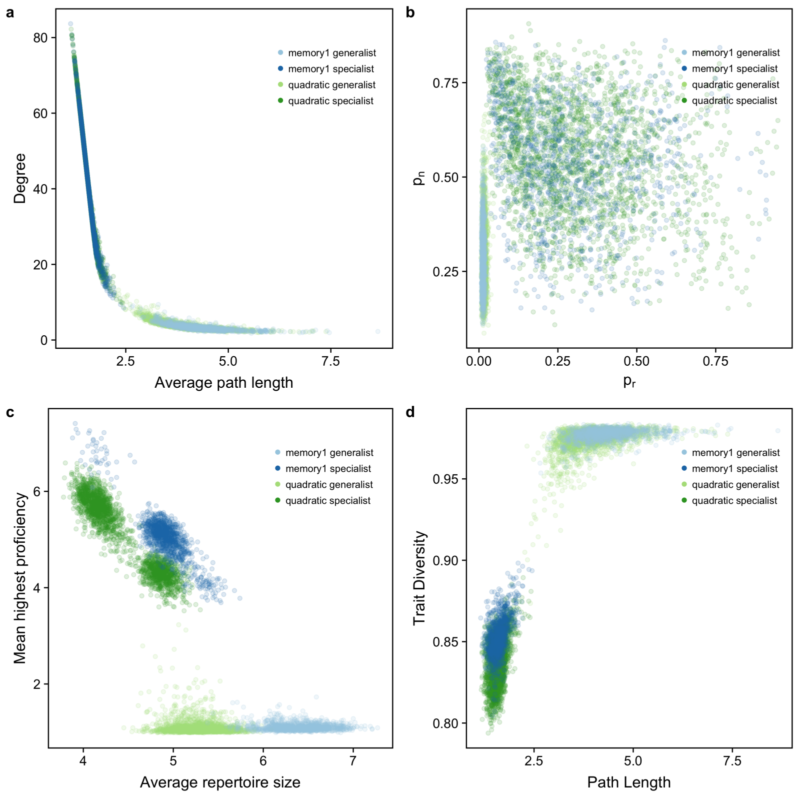

Fig. S2. If memory size is limited, then the two different social learning algorithms are qualitatively the same. Results shown as in fig. S1 but for memory size $m = 1$. For comparison, we also include the results from the main text (labelled 'quadratic').

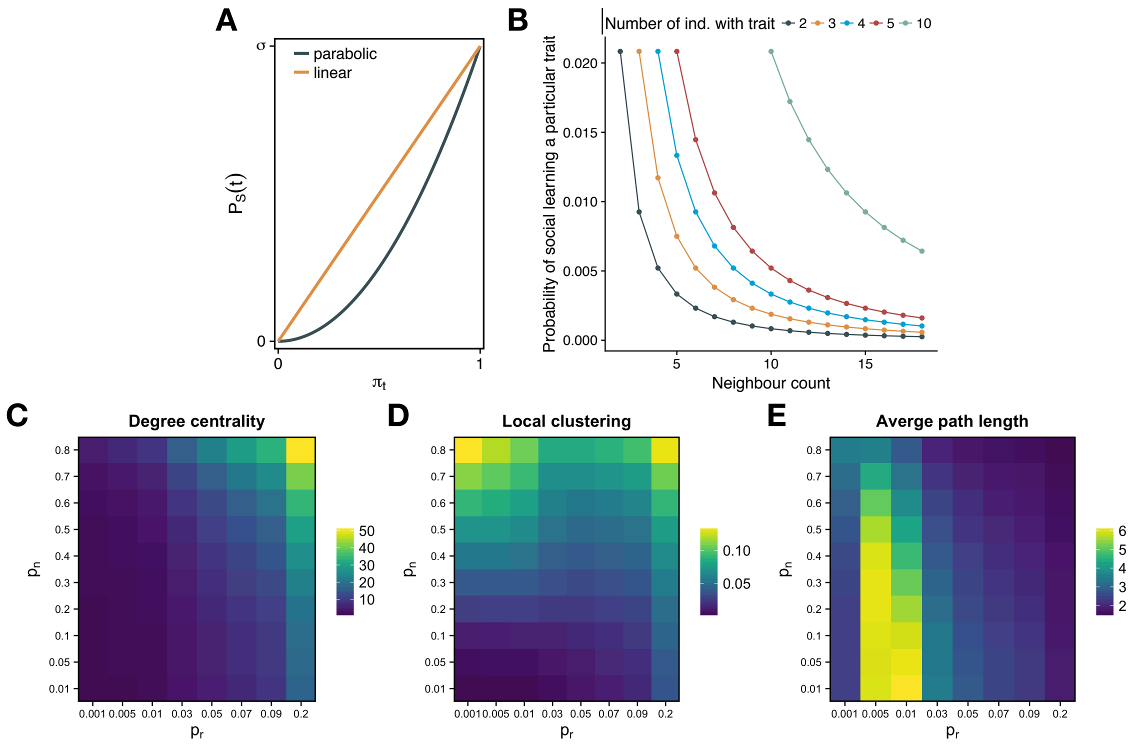

Fig. S3. The effect of complex contagion on social learning dynamics, and of linking parameters on network characteristics. (**A**) Comparing the probability for successful social learning in the linear case ($P_S(t) = \sigma\pi_t$) and the parabolic case ($P_S(t) = \sigma\pi_t^2$), which we use in our model. (**B**) How does the probability to learn a specific trait socially ($P_S(t)$ on y-axis) change as number of neighbours (x-axis) and number of individuals with this trait (n_{it}) changes (all neighbours with repertoire size 6). Network metrics for fixed p_n and p_r. **C**, Degree centrality is highest for high p_n and p_r. **D**, Clustering is highest for high p_n and p_r (globally clustered network) but also for high p_n and very low p_r (small, isolated local clusters). **E**, Path length is longest for low p_n and low p_r. Data shown here are from the same simulations as results shown in Fig. 2 of the main text.

Section S4. Coupling p_r to p_n to limit degree centrality

In the main text we coupled p_r with p_n to achieve specific average degree centrality ($k \in \{2, 6, 10\}$). To do this, we use the following equation to calculate the value for p_r given p_n that a new individual inherits from its parent, degree k, and graph size N

$$p_r = \frac{k(N-1-p_n(N-2)) - N + 1}{(N-2)(N-1-k)}$$

This equation has been taken directly from ref (*64*). Note that for certain combinations of population size N, degree k, and p_n becomes negative. For example, for $k = 4$ and $N = 100$ for values

$p_n > 0.778\, p_r$ becomes negative. We can calculate for which values of p_n (given k and N) $p_r \leq 0$

$$p_n = \frac{N+1}{N-2} - \frac{N-1}{k(N-2)}$$

which simplifies to

$$p_n = \frac{k(N+1) - (N-1)}{k(N-2)}$$

We set $p_r = 0$ whenever $p_r < 0$ in our simulations.

In fig. S4 and S5 we compare evolved networks for three different average degrees ($k \in \{2, 6, 10\}$) and for the generalist and specialist selection regimes. We find the strongest differences for $k = 6$, where under specialist selection almost all individuals possess the most common trait (fig. S4E), whereas under generalist selection more than three traits are widely distributed throughout the population(fig. S4B). Also, although the average degree is the same we find that under specialist selection almost all individuals achieve trait proficiencies above 2 (fig. S5E), whereas under generalist selection fewer individuals reach this level (fig. S5B). Results are much more similar for both selection regimes for low ($k = 2$) and high ($k = 10$) degree.

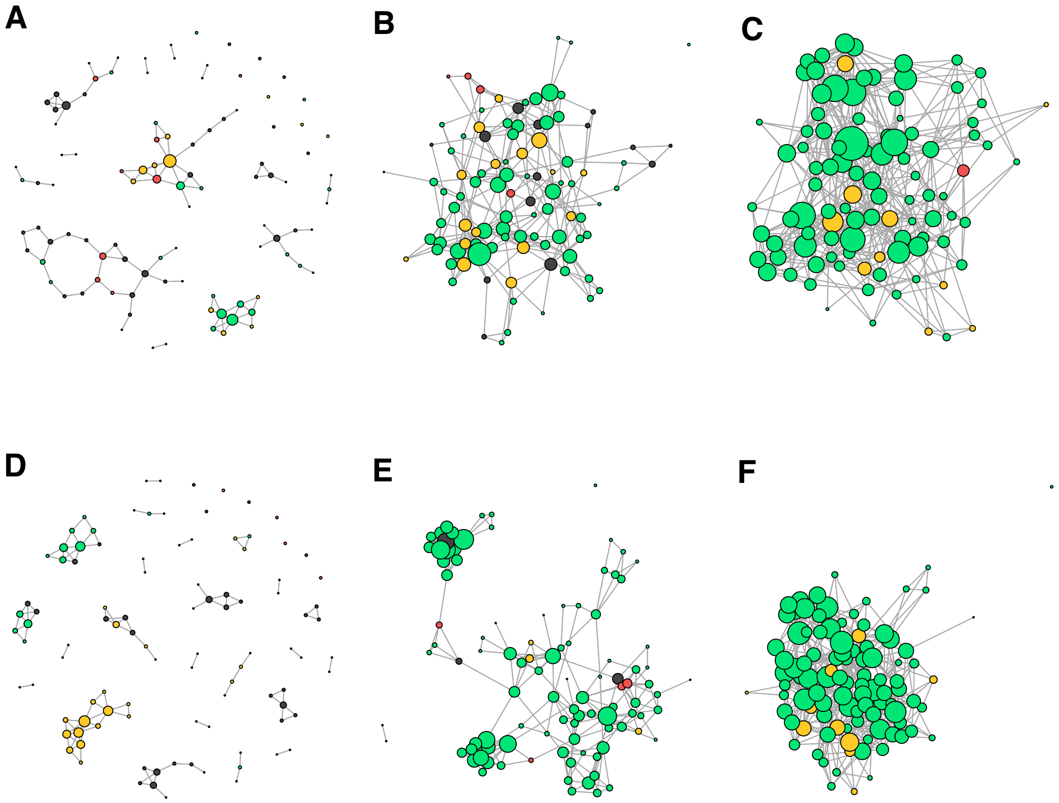

Fig. S4. Distribution of common traits depends on average connectivity. As average connectivity increases from $k = 2$ (**A**,**D**), to $k = 6$ (**B**,**E**), and $k = 10$ (**C**,**F**) the distribution of the most common traits changes both for populations selected for generalist knowledge (**A**-**C**), and specialist knowledge (**D**-**F**). Individuals are coloured based on whether they possess the most common (green), second most common (orange), or third most common (red) trait, or neither of the three most common traits (blue) in that order. The largest difference between selection regimes are at intermediate connectivity (**B**,**E**), where almost all specialists posses the most common traits, whereas generalists form clusters of different knowledge sets. Note, some individuals have more than k connections (due to giving birth), or less than k connections (death of a neighbour).

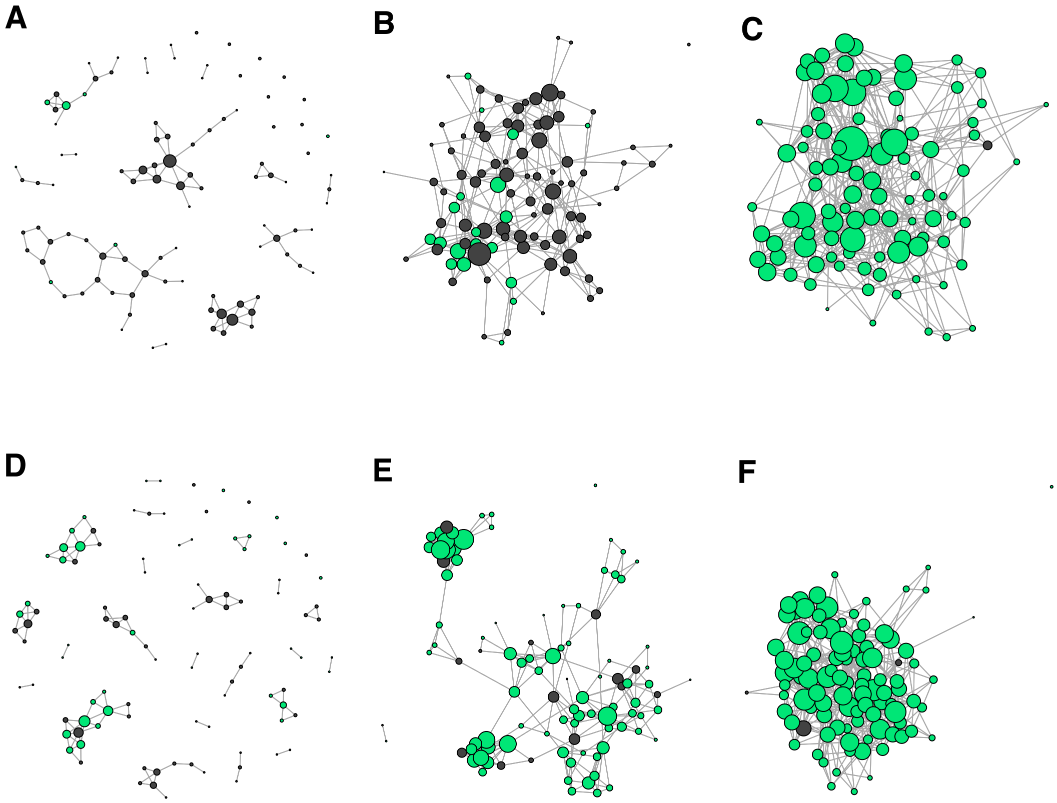

Fig. S5. Trait proficiency depends on the level of trait convergence and connectivity. Shown are the same networks as in fig. S4, however, nodes are green if at least one trait proficiency in the individual's repertoire is larger than two, otherwise they are blue. Similar to fig. S4 we find the largest differences for intermediate connectivity (**B**,**E**) where almost all specialists have a proficiency higher than two, whereas almost all generalists have a lower proficiency.

Section S5. Time series for simulations with evolving p_n and p_r

In fig. S6 we combine the trajectories of the linking probabilities p_n and p_r for all three selection regimes (neutral, specialist, generalist). Here, we can see that under specialist selection p_n but also p_r increases faster than when selection is neutral.

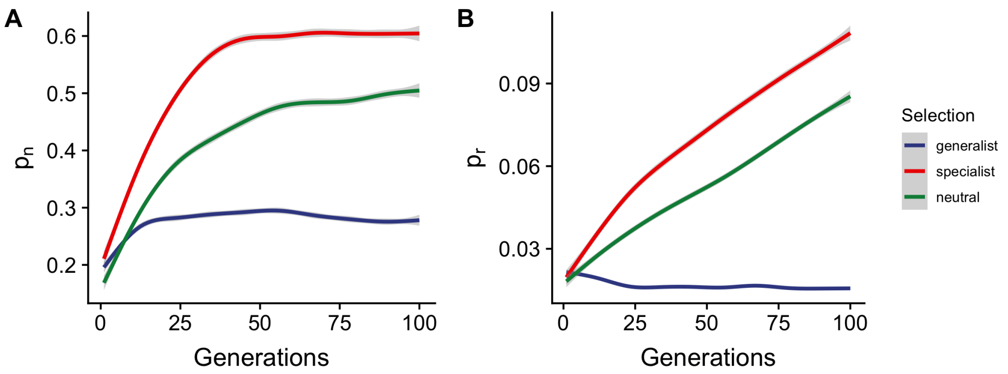

Fig. S6. Trajectories for linking probabilities p_n and p_r averaged over all simulation runs for all three selection regimes (neutral, generalist, and specialist). **A**, p_n increases strongest and fasted under specialist selection. **B**, p_r drops for generalist selection. Data shown here are from the same simulations as data shown in Fig. 2 in the main text.

Section S6. Low mutation rate

Results in the main text are from simulations with mutation rate $\mu = 1$. Here, we show that at a lower mutation rate $(\mu = 0.01)$ our results remain qualitatively unchanged (fig. S7).

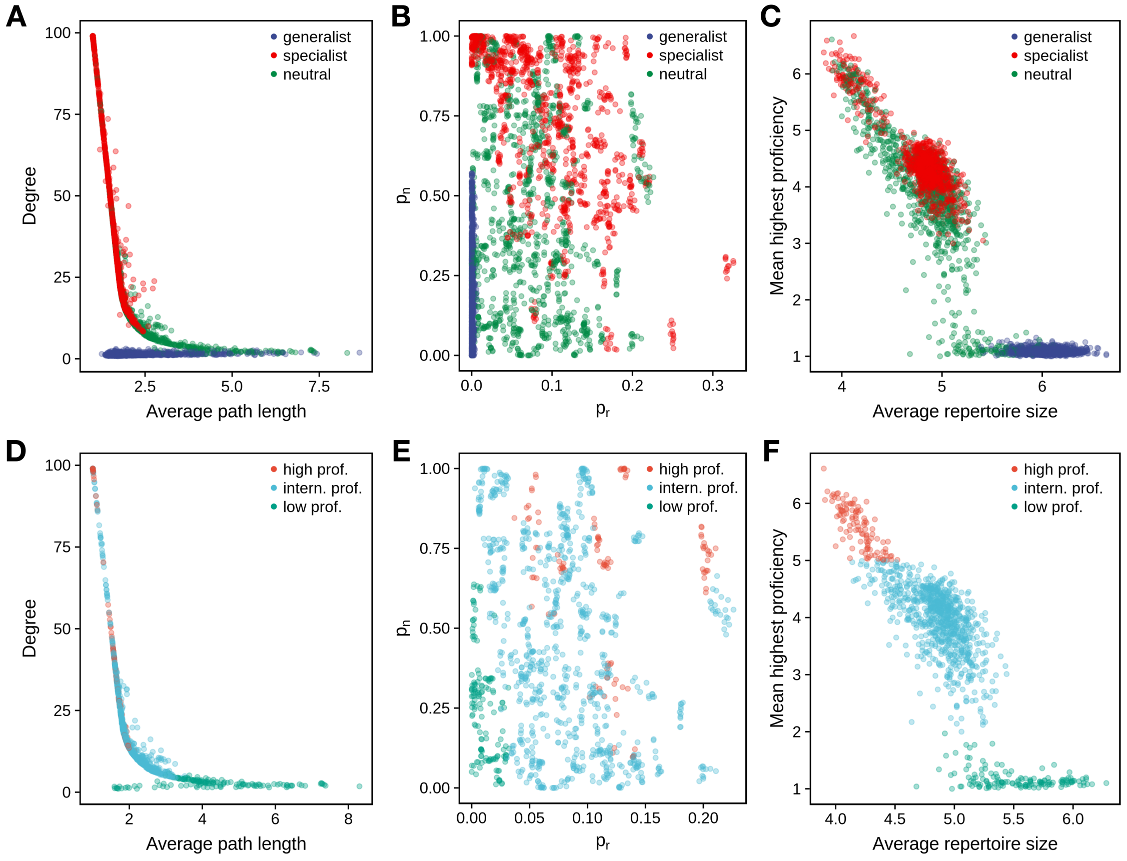

Fig. S7. Results displayed as in Fig. 2 of the main text but with mutation rate μ = 0.01. Overall, results are comparable. (**A–C**) However, we find that some specialist populations have formed complete graphs (degree 100, **a**). Also, it is apparent that generalist networks have shorter average path lengths then in Fig. 2. This is because with the lower mutation rate p_r can be much closer to 0 and so generalists are more likely to form isolated clustered (where path length are short). Populations under neutral selection are between both selection regimes. **(D-F)** For better visualisation we plotted here only results for neutral selection from **A-B**. Colours are chosen to indicate high (> 5), low (< 2), and intermediate proficiency (**D**), and how proficiency relates to the linking probabilities (**E**).

Section S7. Connection costs

In the main text, we assume that forming and maintaining connections comes at no extra cost to the individual, other than diluting the signal that might come from its neighbours. Here, we subtract a fixed cost per connection from an individual's payoff and observe how this is changing connectedness. We find that connection costs mainly affect specialists as they usually form dense networks. By adding connection costs specialists form fever connections. Thus, more sparsely connected networks with longer average path length emerge (fig. S8A), which reduces average

trait proficiency but increases repertoire size (fig. S8B).

For comparison, we include figures in the same style as Fig. 2 and 5 but with connection costs. The results remain qualitatively similar. However, we find that p_n and p_r are not drifting as much under specialist selection as we have seen without connection costs (fig. S9A–C). Also, when switching from specialist to generalist selection there are fewer populations that do not return to a more sparsely connected state than what we observe without connection costs (fig. S9D-G). In conclusion, adding connection costs avoids that the population is drifting towards a fully connected graph, which allows the population to increase trait diversity more easily once selection pressure changes.

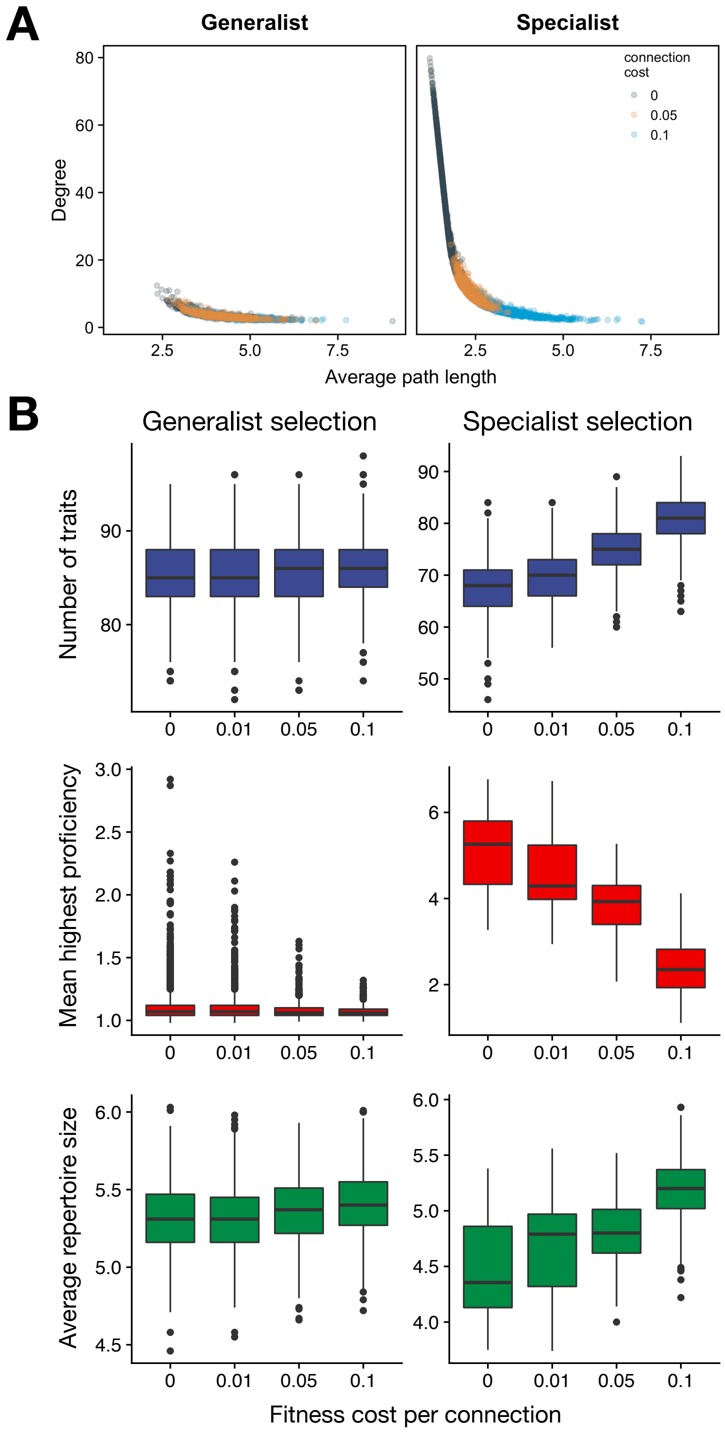

Fig. S8. Adding a cost per connection reduces average degree in specialists, whereas generalists are less affected. (**A**) Shown are average degree and path length for simulations with connection costs and for generalist (left) or specialist (right) selection. Generalists are less affected by connection costs, as they generally form fewer connections than specialists. High connection costs makes networks more similar to those under generalist selection. (**B**) Increasing connection costs makes populations more similar to those under generalist selection (left column), even if they are under specialist selection (right column).

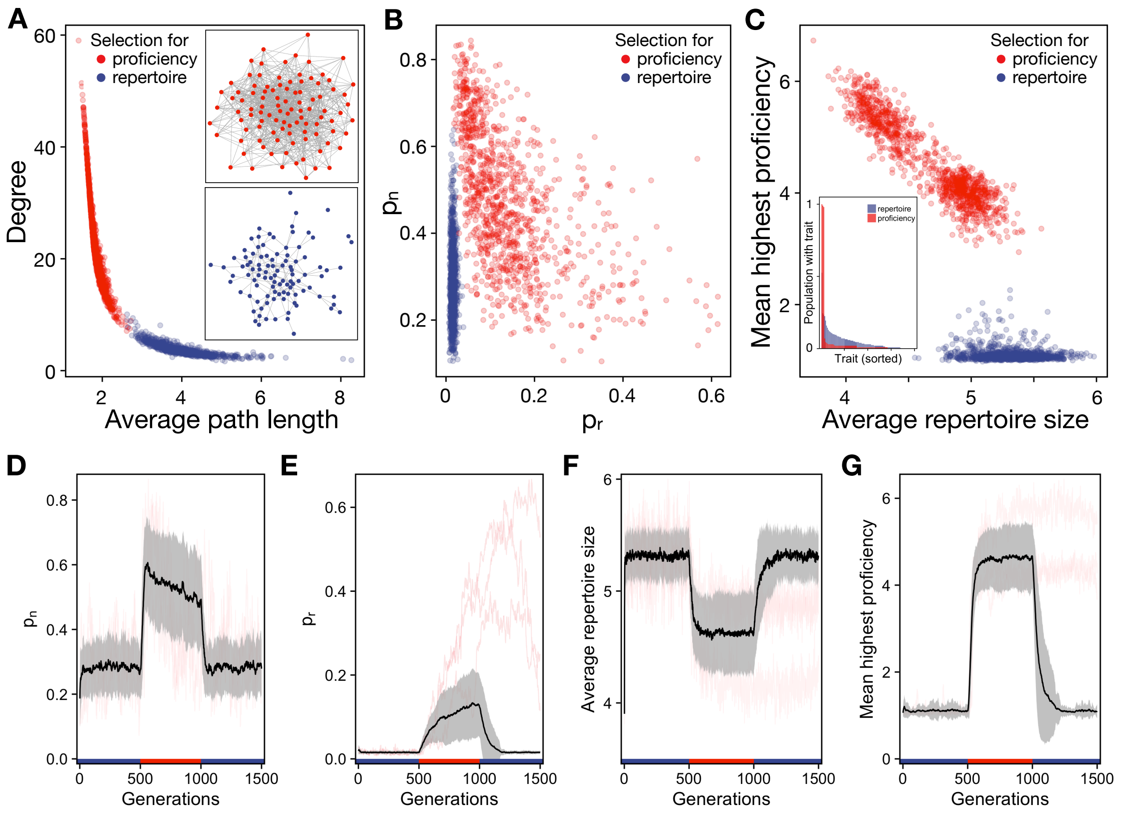

Fig. S9. Added connection costs. (**A-C**) Data are presented as in Fig. 3 in main text but with fitness cost 0.01 per connection. The results are qualitatively similar, however, we find less drifting of linking parameters and overall lower average degree for specialists. (**D-G**) Data are presented as in Fig. 5 in main text but with fitness cost 0.01 per connection. The results are qualitatively similar, however, we find that fewer simulations fail to switch from specialist to generalist selection (red lines).

Section S8. Varying population size and trait number

In the main text, we presented data for simulations with $N = 100$ individuals and $T = 100$ traits. However, we also ran simulations for different population sizes ($N \in \{10^1, 10^{1.5}, 10^2, 10^{2.5}, 10^3\}$)

and different number of traits ($T \in \{50, 100, 150, 200, 250\}$). All other parameters are identical to those used for simulation in Fig. 3. We find that social inheritance (p_n) and random linkage (p_r) is consistently high under specialist and neutral selection, whereas both parameters are low for generalists (fig. S10A,B). Interestingly, we find that specialists (and neutral selection) achieve the average highest proficiency for large populations and a large number of traits (fig. S10C). The reason for this is that the higher possible number of traits in these worlds makes it less likely that innovation events among neighbours result in the same trait. Hence, rare traits are even less likely to be learned and overall repertoire sizes remain smaller (fig. S10D). Consequently, we also find the lowest number of possible traits known to a population in those simulations where there are many traits available (i.e. 10^3 traits, fig. S10E). Overall, specialists seem to benefit from larger populations but also from a wider variety of possible traits (which undermine the effect of innovating novel traits). Generalists on the other side do best either if the number of traits is small or if populations are small (fig. S10D).

As an example, we plotted results for $T = 100$ from fig. S10 in fig. S11. Two results stand out. First, we find that results from the end of specialist and generalist simulations look very similar. As we have shown, these two selection regimes are different at the initial stage but are both drifting towards fully connected networks.

Second, we find that even with larger populations both average repertoire size and average highest proficiency plateau (fig. S11B,C). As we describe in the main text, this is due to the limit number of learning turns individuals have to acquire traits. There are 100 learning turns, and (as in the main text) we assume a social learning success rate of $\sigma = 0.75$, which would allow a maximum proficiency of about 8. This is only true if all neighbours only have a repertoire of size 1 and all share the same trait. However, even in the larger populations repertoire size is not smaller than 4, which means that occasionally a learning turn is used to improve proficiency in a different trait.

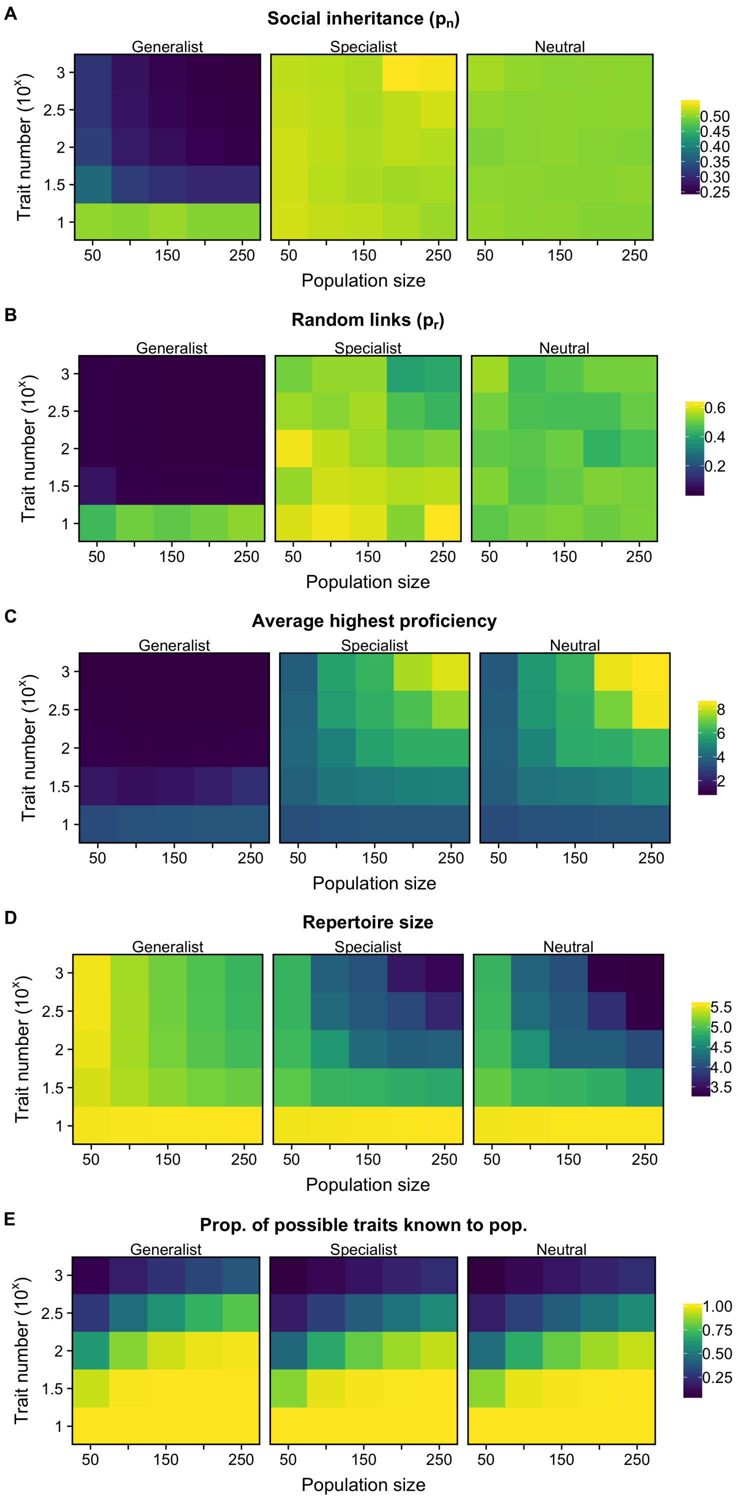

Fig. S10. Varying the number of traits and individuals in a population. Each square represents the average of 100 simulations for generalist, specialist, and neutral selection. Otherwise, parameters are identical to those used for Fig. 3.

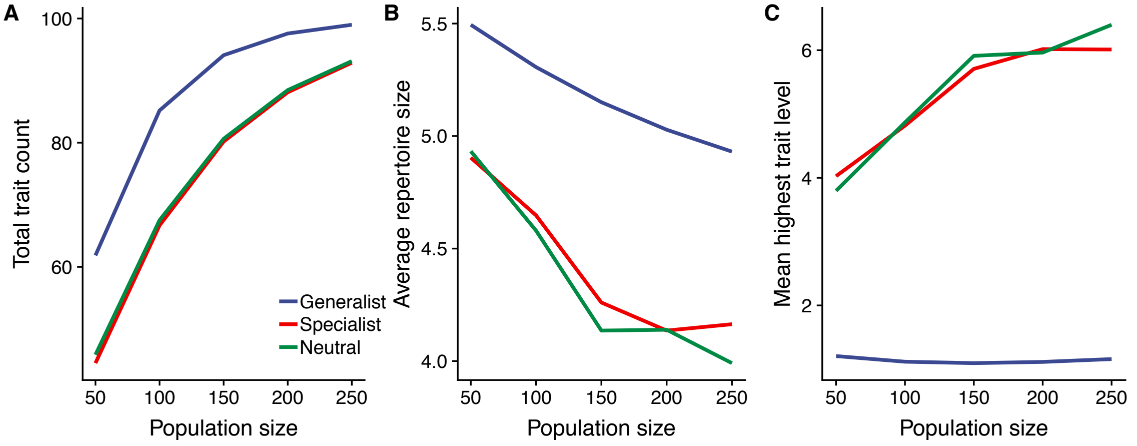

Fig. S11. Increasing population size also increases trait diversity in the population. Exemplary, the figure shows data from fig. S10 for 100 traits. We find that both the total trait diversity (**A**), as well as trait proficiency (**C**) increases with population size. As expected, higher proficiency coincides with smaller repertoires (**B**).

Section S9. Varying innovation and social learning success rate

Throughout the main text, we used social learning success rate 0.75 and an innovation success rate of 0.01, per learning attempt. For fig. S12, we systematically vary both rates and observe how this affects the results.

In general, we find that trait diversity in populations is higher for higher rates of innovation (fig. S12E), independent of selection regime and social learning rate. We also find only small differences in repertoire size, except for very high innovation rates (fig. S12D). Interestingly, generalists do best when both rates are high, as this provides large trait diversity and high chances to acquire these traits. Conversely, while specialists benefit from high social learning success rates, high innovation rates undermine local (and by extension, global) trait convergence, which makes it less likely to engage repeatedly with the same traits (fig. S12C). For linking parameters, we find that parameters drift for all combinations of innovation and copying rates under specialist and neutral selection (fig. S12A,B). However, for generalists, this is only true where there is no social learning (here, the social network does not matter) and where innovation rates are very low (here, populations converge on traits as there is not a sufficient number of traits innovated).

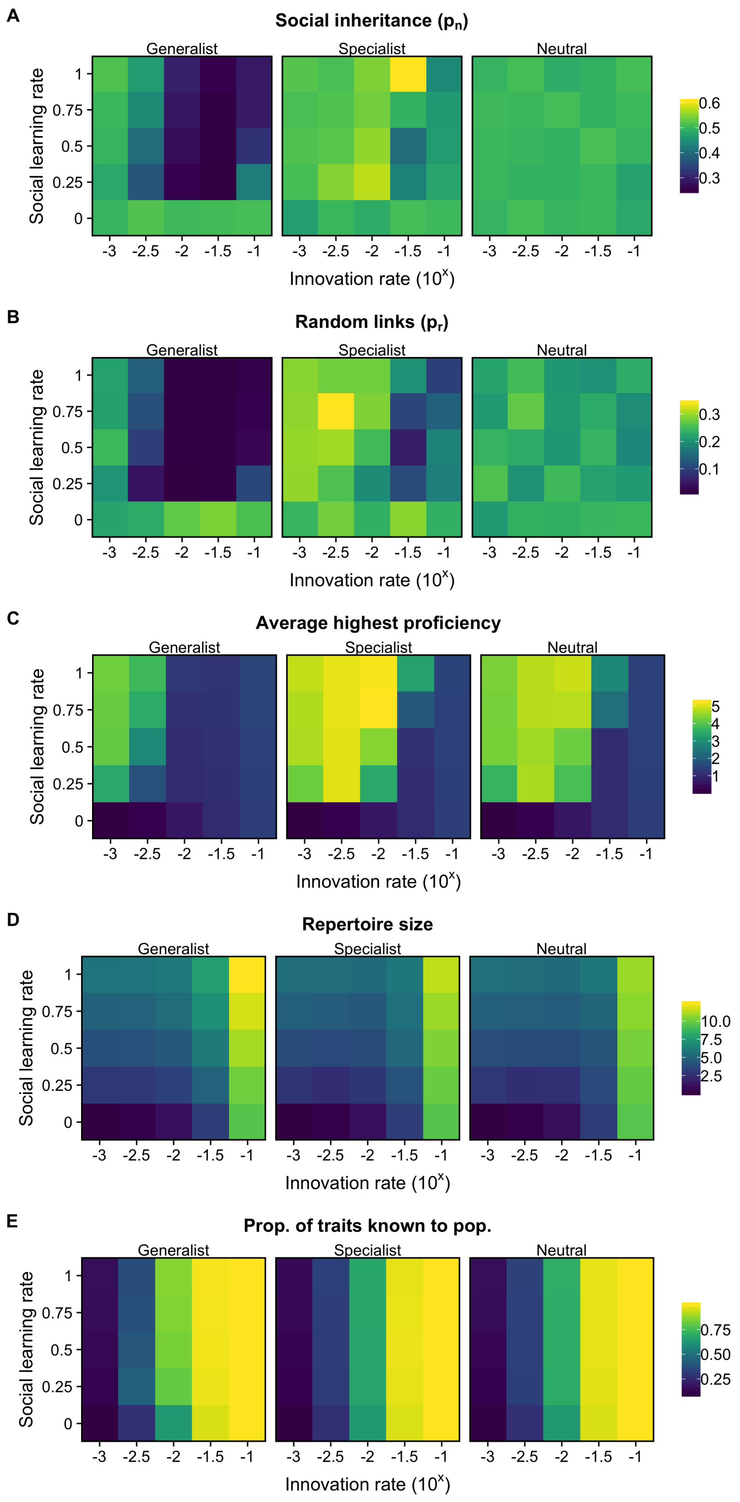

Fig. S12. Varying innovation and social learning success rate. Each square represents the average of 100 simulations for generalist, specialist, and neutral selection. Otherwise, parameters are identical to those used for Fig. 3.
